# Comparative effectiveness of radiotherapy for early‐stage hormone receptor‐positive breast cancer in elderly women using real‐world data

**DOI:** 10.1002/cam4.1904

**Published:** 2018-12-12

**Authors:** Askal A. Ali, Rima Tawk, Hong Xiao, Ellen Campbell, Anastasia Semykina, Alberto J. Montero, Muluberhan Mogos, Vakaramoko Diaby

**Affiliations:** ^1^ Economic, Social & Administrative Pharmacy, College of Pharmacy and Pharmaceutical Sciences Florida A&M University Tallahassee Florida; ^2^ Institute of Public Health, College of Pharmacy and Pharmaceutical Sciences Florida A&M University Tallahassee Florida; ^3^ Pharmaceutical Outcomes & Policy (POP), College of Pharmacy University of Florida Gainesville Florida; ^4^ Department of Economics Florida State University Tallahassee Florida; ^5^ Cleveland Clinic Department of Solid Tumor Oncology Taussig Cancer Institute Cleveland Ohio; ^6^ Department of Women, Children and Family Health Science University of Illinois at Chicago Chicago Illinois

**Keywords:** comparative effectiveness, early‐stage hormon receptor positive breast cancer, elderly women, radiotherapy, real‐world data

## Abstract

**Background:**

Radiotherapy is the recommended treatment after breast‐conserving surgery (BCS) for early‐stage breast cancer (BC). However, there is no clear evidence whether radiotherapy after BCS improves the survival of elderly women diagnosed with early‐stage hormone receptor‐positive (HR+) BC. The aim of this study was to investigate the survival benefit associated with radiotherapy plus hormonal therapy vs hormonal therapy alone after BCS for early‐stage HR+ BC patients.

**Methods:**

Using the Surveillance, Epidemiology, and End Results linked with Medicare data, we identified elderly (65 years and older) women diagnosed with early‐stage HR+ BC (2006‐2011) who received hormonal therapy with or without radiotherapy after BCS. A log‐rank test, Cox proportional hazards models, and propensity score matching were used to estimate the overall survival (OS) benefit associated with radiotherapy after BCS.

**Results:**

Of the 5688 patients, there were 303 deaths from any cause. One hundred and eighty‐five (61%) of these deaths occurred in the hormonal therapy group, and 118 (39%) deaths occurred in the radiotherapy plus hormonal therapy group. The mean survival time in the radiotherapy plus hormonal therapy group was 5.32 ± 1.86 years compared with 4.92 ± 1.86 years in the hormonal therapy group. Based on the adjusted and propensity score matching analysis, patients in the adjuvant radiotherapy group had a lower risk of death compared with those who did not receive radiotherapy. Radiotherapy plus hormonal therapy decreased the risk of death by 32%. The effect estimates were similar in the adjusted and matched cohorts.

**Conclusions:**

Radiotherapy plus hormonal therapy resulted in a significant improvement in the OS of elderly women diagnosed with HR+ BC.

## 1 INTRODUCTION

Radiotherapy is the recommended treatment after breast‐conserving surgery (BCS) for early‐stage breast cancer (BC). However, it is not clear whether the addition of radiotherapy after BCS results in improved survival in elderly women diagnosed with early‐stage hormone receptor‐positive (HR+) BC. The use of radiotherapy in elderly women has decreased following the Cancer and Leukemia Group B (CALGB) C9343 trial's conclusion that adjuvant radiotherapy did not improve survival in elderly patients with HR+ BC.^1^ Subsequent studies have reported similar findings.^2^, ^3^ Based on these trial results, the National Comprehensive Cancer Network (NCCN) guidelines recommend that adjuvant radiotherapy be omitted for patients 70 years and older with T1N0M0 ER+HER2‐negative BC.^9^ Conversely, findings from observational studies showed that the addition of radiotherapy after BCS was associated with improved survival of elderly women.^10^, ^11^ These studies proposed that radiotherapy be considered a noteworthy aspect of a treatment plan for appropriately selected elderly patients and that age should not be the only factor to consider when making decisions about whether elderly patients with early‐stage BC should receive radiotherapy.^10^, ^11^

Some of the differences between the results of randomized clinical trials (RCTs) and observational studies may derive from the limitations of these studies’ designs. On the one hand, RCTs are often shorter in duration and conducted in controlled environments with highly selected patients; they may not be generalizable to everyday clinical practice characterized by more heterogeneity in patient population, providers, and health care delivery systems.^12^, ^13^ On the other hand, the most important limitation of the nonrandomization of observational studies is selection bias,^14^ which occurs when the treatment and control groups are systematically different from one another. These factors can be associated with the outcome and the choice of treatment.^15^, ^16^

There is accordingly a critical need to generate robust evidence regarding survival benefits associated with radiotherapy after BCS in elderly patients with early‐stage HR+ BC. This evidence can be used to optimize the use of radiotherapy to avoid under or overtreatment and improve clinical decision‐making for elderly patients with early‐stage HR+ BC.

The aim of this study was to estimate the survival benefit associated with radiotherapy plus hormonal therapy (HT) vs HT alone for early‐stage HR+ BC to inform clinical decision‐making.

## 2 METHODS

### 2.1 Study population and data sources

We studied women diagnosed with early‐stage (stage I–IIB) HR+ BC who had undergone surgical treatment identified in Surveillance, Epidemiology, and End Results (SEER)‐Medicare data. The 6th edition of the American Joint Committee on Cancer (AJCC) Cancer Staging Manual was used to identify early‐stage BC. We identified patients who received surgical treatment, radiotherapy, HT, and a combination of radiotherapy and HT. We included data from 2006 to 2011 pertaining to women aged 65 and older at diagnosis who were enrolled in Medicare Part A and Part B. Patients who enrolled in Health Maintenance Organizations (HMOs) and patients diagnosed through autopsy or by death certificate were excluded.

### 2.2 Treatment, outcome, and covariates

We compared radiotherapy plus HT vs HT alone after BCS. Adjuvant systemic treatments were identified as the receipt of radiotherapy using Healthcare Common Procedure Coding system codes (HCPCS), International Classification of Diseases, Ninth Revision Clinical Modification (ICD‐9‐CM) codes, brand name (bn) or generic name (gn) or “Tamoxifen Citrate” for Tamoxifen to identify HT status. See Table S1 for detailed treatment identification. Overall survival (OS) times were calculated as “number of years” from the date of diagnosis to the date of death or termination of the study. Death from all causes was the event of interest.

Patient demographics and clinical and area‐level characteristics were obtained from the Patient Entitlement and Diagnosis Summary File (PEDSF). Charlson comorbidity scores were derived from Medicare Provider Analysis and Review (MEDPAR), National Claims History (NCH), and outpatient files during the 12 months preceding the diagnosis utilizing ICD‐9 codes. Hospital characteristics were identified from the hospital file.

### 2.3 Statistical analysis

The baseline characteristics of patients receiving radiotherapy plus HT and HT alone were compared using the Student's *t* test for continuous variables and the chi‐squared test for categorical variables assuming equal variance. Because the treatment assignments were not random, propensity score (PS) matching analysis was performed. The use of PS matching ensures equivalence in the distribution of observed confounders between the two treatment groups. A two‐stage PS matching approach was adopted. In the first stage, we estimated the probability of receiving treatment (PS) given the observed covariates using a logistic model (see Table S2). We matched the control group (HT alone) with the treatment group (radiotherapy plus HT) after BCS using a one‐to‐one greedy matching procedure with a caliper equal to one‐fifth of the standard deviation (SD).^17^ The second stage consisted of estimating the survival benefit.

The balance in baseline covariates between the HT alone and radiotherapy plus HT groups was assessed using graphical and analytical approaches. We plotted and compared the distribution of PS for the HT alone and radiotherapy plus HT groups before and after matching. The overall quality of matching was assessed by calculating the absolute standardized differences (ASDs) for each baseline covariate. We compared the pseudo *R* ^2^ for unmatched and matched data from the logistic regression.

After PS matching, survival was assessed using Kaplan‐Meier survival curves; group differences were compared using the log‐rank test. Cox proportional hazard (PH) models were fit to determine any association between radiotherapy plus HT and patient survival. Hazard ratios, *P*‐values, and 95% confidence intervals were calculated. The event of interest in this study was all‐cause death. The observation times were censored at the study calendar time (December 31, 2014) for patients who were alive at the end of the study. Before applying the Cox PH models, we tested the proportionality assumption by visual inspection and analytical tests. We used the Stata*stphplot* graphical test to plot the log‐log transformation of the survival function.^18^, ^19^ The scaled Schoenfeld residuals test for PH assumption was used as an analytical test.^20^ We used time‐dependent variables for the parameters that violated the PH assumption. All of the data were deidentified such that individual patient health information was untracked. An institutional review board exemption was obtained before the initiation of the study. All of the analyses were performed using the SAS version 9.4 and STATA version 14.0 statistical software packages (SAS Institute Inc., Cary, NC, USA).

## 3 RESULTS

### 3.1 Patient characteristics before PS matching

**Figure 1:**
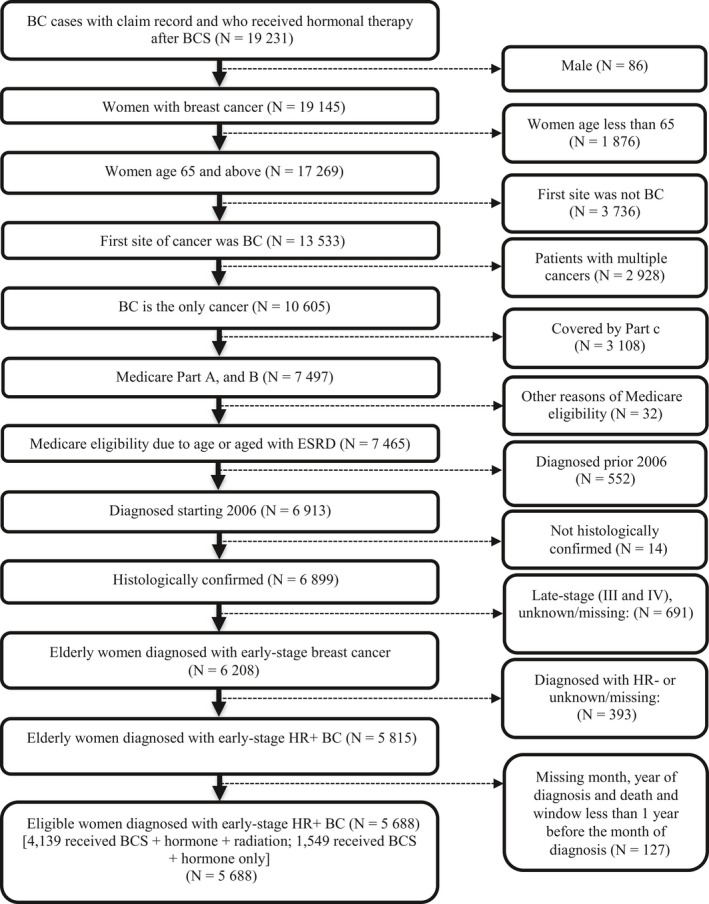
Summary of data extraction. BC, breast cancer; BCS, breast‐conserving surgery; HR+, hormone receptor positive; HR−, hormone receptor negative; N = number of records

A total of 5688 patients satisfied the inclusion criteria (Figure 1). Within this total group, 1549 patients (27.23%) were treated with HT and 4139 patients (72.77%) were treated with radiotherapy plus HT after BCS.

Patient characteristics, facility characteristics, and area‐level information by treatment type are listed in Table 1. The average age at diagnosis was 72.7 years (SD = 5.89 years). Patients who were treated with HT alone after BCS were relatively older than patients who were treated with radiotherapy plus HT (74.81 years vs 72.05 years; *P* < 0.0001). Most of the patients were white (87.05%), and there were several important differences between the HT and the radiotherapy plus HT groups. A large proportion of patients who received HT alone after BCS were single; married women tended to receive radiotherapy plus HT. Patients who were treated with radiotherapy plus HT tended to be diagnosed with a moderately differentiated tumor grade (49.38%), a tumor between 0 and 2 cm in size (81.31%), and no positive lymph node involvement (79.06%). The majority of the women in the radiotherapy plus HT and HT‐only groups had no comorbidities. Among women treated with radiotherapy, 79.06% had no major comorbidities. Among those with known HER2 status, a larger proportion of patients who received radiotherapy plus HT were diagnosed with HER2‐negative BC. Patients who received HT alone had a greater proportion of node‐positive BC compared with patients who received RT (*P* < 0.0001).

**Table 1:** Descriptive characteristics of the study patients by treatment group

Characteristics	Women with breast cancer (N = 5688)	Breast cancer cases by treatment type		
		No radiation (N = 1549)	Radiation (N = 4139)	*P*‐value
Death				
Breast	47 (0.83)	28 (1.81)	19 (0.46)	<.0001
All cause	303 (5.33)	185 (11.94)	118 (2.85)	<.0001
Age in years, N (%)				
Mean age at diagnosis, mean (SD)	72.80 (5.89)	74.81 (6.78)	72.05 (5.18)	<.0001
Between 65 and 69	2016 (35.44)	416 (26.86)	1600 (38.66)	
Between 70 and 74	1650 (29.01)	401 (25.89)	1249 (30.18)	
Between 75 and 79	1243 (21.85)	341 (22.01)	902 (21.79)	
80 and above	779 (13.70)	391 (25.24)	388 (9.37)	
Race, N (%)				
White	4946 (87.05)	1304 (84.29)	3642 (88.08)	0.0024
Black	353 (6.21)	117 (7.56)	236 (5.71)	
Others	383 (6.74)	126 (8.14)	257 (6.22)	
Marital status, N (%)				
Married	2892 (50.84)	661 (42.67)	2231 (53.90)	<.0001
Unmarried	2532 (44.51)	806 (52.03)	1726 (41.70)	
Unknown	264 (4.64)	82 (5.29)	182 (4.40)	
Charlson comorbidity				
0	3546 (62.34)	835 (53.91)	2711 (65.5)	<.0001
1‐3	2007 (35.28)	658 (42.48)	1349 (32.59)	
≥4	135 (2.37)	56 (3.62)	79 (1.91)	
Grade, N (%)				
Well differentiated	1772 (31.15)	483 (31.18)	1289 (31.14)	0.1494
Moderately differentiated	2771 (48.72)	727 (46.93)	2044 (49.38)	
Poorly differentiated and undifferentiated	879. (15.45)	255 (16.46)	624 (15.08)	
Unknown/inapplicable	266 (4.68)	84 (5.42)	182 (4.40)	
Tumor size, N (%)				
0‐2 cm	4509 (79.27)	1151 (74.31)	3358 (81.31)	<.0001
2‐5 cm	1020 (17.93)	351 (22.66)	669 (16.16)	
≥5 cm	159 (2.80)	47 (3.03)	112 (2.71)	
Number of positive lymph nodes, N (%)				
0	4357 (76.65)	1087 (70.22)	3270 (79.06)	<.0001
1‐3	875 (15.39)	260 (16.80)	615 (14.87)	
≥4	452 (7.95)	201 (12.98)	251 (6.07)	
HER2, N (%)				
Positive	113 (1.99)	35 (2.26)	78 (1.88)	0.5732
Negative	1418 (24.93)	393 (25.35)	1025 (24.76)	
Unknown	4157 (73.08)	1121 (72.37)	3036 (73.35)	
Year of diagnosis, N (%)				
2006	1647 (28.96)	397 (25.63)	1250 (30.20)	0.0241
2007	815 (14.33)	243 (15.69)	572 (13.82)	
2008	839 (14.75)	237 (15.30)	602 (14.54)	
2009	768 (13.50)	217 (14.01)	551 (13.31)	
2010	819 (14.40)	237 (15.30)	582 (14.06)	
2011	800 (14.06)	218 (14.07)	582 (14.06)	
Metropolitan area, N (%)				
Yes	5030 (88.43)	1272 (82.12)	3758 (90.97)	<.0001
No	658 (11.57)	277 (17.88)	381 (9.21)	
Geographic regions, N (%)				
North East	1348 (23.70)	293 (18.92)	1055 (25.49)	<.0001
Midwest	752 (13.22)	192 (12.40)	560 (13.53)	
South	1322 (23.24)	496 (32.02)	826 (19.96)	
West	2266 (39.84)	568 (36.67)	1698 (41.02)	
Poverty level (%)				
0‐5	1703 (29.99)	342 (22.12)	1361 (32.93)	<.0001
5‐10	1568 (27.61)	389 (25.16)	1179 (28.53)	
10‐20	1511 (26.60)	473 (30.60)	1038 (24.12)	
20‐100	897 (15.80)	372 (22.12)	555 (13.43)	
Referral hospital, N (%)				
Yes	254 (4.47)	98 (6.33)	156 (3.77)	<.0001
No	3120 (54.85)	1009 (65.14)	2111 (51.00)	
Unknown	2314 (40.68)	442 (28.53)	1872 (45.23)	
Survival time year				
Mean				
Overall survival	5.22 (1.86)	4.92 (1.84)	5.32 (1.86)	<.0001

HER2, human epidermal growth factor receptor; N, number of records; (%), column percentage.

During the study period, 303 deaths occurred. There were 185 (11.94%) deaths among patients treated with HT alone, and 118 (2.85%) deaths among patients treated with radiotherapy plus HT (*P* < 0.0001). The mean OS time was 5.22 years (SD = 0.01 years). Compared with patients treated with HT alone, patients treated with radiotherapy plus HT had a relatively longer average survival time (4.92 years vs 5.32 years; *P* < 0.0001).

### 3.2 PS matching

Nine hundred and thirty‐six of the 4218 radiation‐treated patients were matched with 936 of the 1591 HT patients. The numbers of deaths from any causes in the HT and radiotherapy plus HT groups were 106 and 67, respectively. The mean survival time after PS matching for the radiotherapy plus HT group was 5.18 ± 1.80 years; the mean survival time in the HT group was 5.10 ± 1.80X years.

The balancing property of PS matching was satisfied with six optimal final numbers of blocks. Table S2 lists the distribution of patient baseline characteristics before and after PS matching. There were no statistically significant differences in baseline patient characteristics after PS matching.

**Figure 2:**
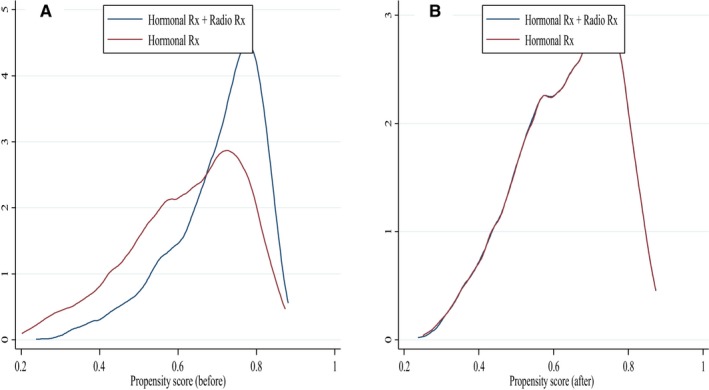
Propensity score density graph for the treatment groups. A, Propensity score density graph for treatment groups before matching; B, Propensity score density graph for treatment groups after matching. Hormonal Rx + Radio Rx = HT plus radiotherapy (blue line); Hormonal Rx = HT alone (red line)

Figure 2 shows a nonparametric density estimate of the distribution of the estimated PS before and after matching for each patient group. The estimated ASDs are <0.1 (see Table S3), and the *R* ^2^ was reduced to 0.0053 from 0.0746 after matching. Consequently, the balancing assumption was successfully satisfied.

### 3.3 Survival analysis

**Figure 3:**
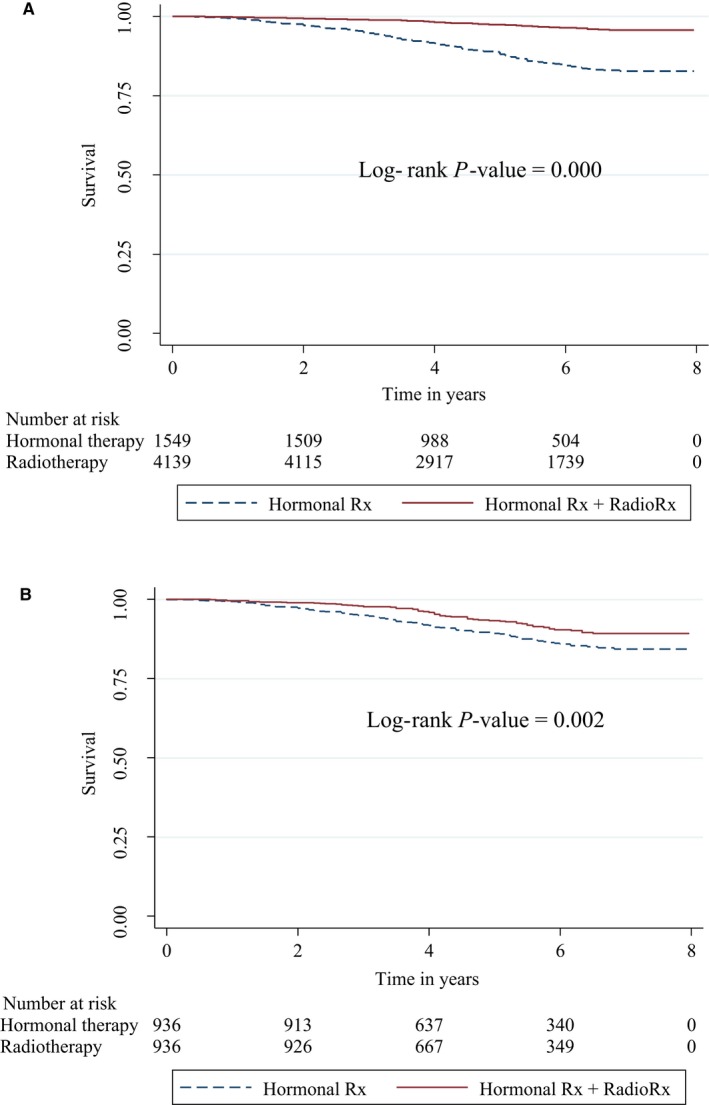
Kaplan‐Meier estimates of OS. A, Kaplan‐Meier estimates of OS for all patients (before PS matching); B, Kaplan‐Meier estimates of OS for matched patients with HT vs radiotherapy plus HT. Hormonal Rx + Radio Rx = HT plus radiotherapy (red line); Hormonal Rx = HT alone (dashed blue line). The *y* axis is the probability of survival, and the *x* axis is time to survival in years

Figure 3 shows the Kaplan‐Meier curve and compares the OS for the radiotherapy plus HT and HT alone after BCS groups. The unadjusted analysis indicates that patients who underwent radiotherapy plus HT had a higher OS rate compared with patients treated with HT alone after BCS (*P* < 0.0001).

Prior to the PS matching, the graphical test revealed survival curves that were not parallel, suggesting that the PH assumption condition was likely to be violated (Figure S1A). The analytical test (scaled Schoenfeld residuals test) for the PH assumption indicated that age at diagnosis (*P*‐value < 0.000) and year of diagnosis (*P*‐value = 0.044) violated the proportionality assumption. Therefore, we used time‐dependent variables for the parameters that violated the PH assumption. After PS matching, the graphical (Figure S1B) and analytical tests confirmed that the PH assumption was satisfied.

Cox PH models were fit to the unmatched and matched datasets. The results of the multivariate Cox PH model for the unmatched data are presented in Table 2. These results suggest that patients who received radiotherapy plus HT after BCS had their risk of death significantly decreased by 32% (hazard ratio [HR] = 0.68; *P*‐value = 0.012) compared with patients who received HT alone. There was no statistically significant difference in OS as a function of race.

**Table 2:** Multivariate Cox proportional hazards regression predictors of overall survival (N = 3050)

	Hazard. ratio	95% confidence interval		*P*‐value
		Lower	Upper	
Treatment				
RadioRx + Hormonal Rx vs Hormonal Rx	0.683	0.508	0.918	0.012
Age at diagnosis				
Age 70 and 74 vs Age 65 and 69	0.241	0.049	1.202	0.083
Age75 and 79 vs Age 65 and 69	0.078	0.009	0.662	0.019
Age 80+ vs Age 65 and 69	13.495	4.507	40.408	0
Race				
Black vs White	1.540	0.882	2.689	0.129
Other vs White	0.758	0.379	1.514	0.433
Marital status				
Married vs not married	0.945	0.682	1.309	0.733
Charlson comorbidity				
1‐3 vs 0	1.604	1.191	2.159	0.002
≥4 vs 0	1.841	0.978	3.465	0.059
Tumor Grade				
Moderately differentiated vs Well differentiated	1.119	0.784	1.598	0.536
Poorly differentiated and undifferentiated vs Well differentiated	1.043	0.669	1.626	0.853
Tumor size (cm)				
2‐4 vs <2	1.606	1.174	2.197	0.003
≥ 5 vs <2	1.853	0.846	4.058	0.123
Number of node‐positive				
1‐3 vs 0	1.435	0.958	2.148	0.08
≥ 4 vs 0	1.778	1.282	2.466	0.001
Metropolitan area				
Yes vs No	0.843	0.5184313	1.37004	0.49
Referral hospital				
Yes vs No	0.895	0.547	1.463	0.658
Year of diagnosis				
2007 vs 2006	1.203	0.756	1.913	0.437
2008 vs 2006	0.763	0.358	1.625	0.483
2009 vs 2006	0.817	0.324	2.059	0.668
2010 vs 2006	0.377	0.113	1.260	0.113
2011 vs 2006	0.136	0.017	1.063	0.057
Geographic regions				
Midwest vs Northeast	1.295	0.795	2.109	0.298
West vs Northeast	1.181	0.762	1.828	0.457
South vs Northeast	1.226	0.833	1.805	0.301
Poverty level				
5%‐10% vs 0%‐5%	1.069	0.722	1.583	0.737
10%‐20% vs 0%‐5%	1.034	0.696	1.535	0.87
20%‐100% vs 0%‐5%	1.352	0.871	2.100	0.179
Interaction with ln(time)				
Age	1.774	1.259	2.500	0.001
Year of diagnosis	0.771	0.598	0.993	0.044

The stratum age 80+ was associated with an elevated risk of dying immediately after diagnosis (HR = 13.5, *P* = 0.00) compared with the group of patients diagnosed between the ages of 65 and 69. The interaction between the natural log of time and age at diagnosis suggests that the hazard intensifies with the increasing age of diagnosis. Women with multiple comorbidities had a higher risk of death compared with women with no comorbidities.

Patients who had one or more positive lymph nodes had a higher risk of death compared with women who had no positive lymph nodes. Compared with patients diagnosed with a tumor size <2 cm, patients diagnosed with a tumor size of 2‐4 cm had a shorter survival time. The interaction between year of diagnosis and the natural log of time suggests that the hazard decreases diagnoses made more recently.

In the PS‐matched sample, patients who were treated with radiotherapy plus HT exhibited a 38% lower risk of death compared with patients who had received only HT. Based on the three analyses we conducted, we found a higher risk of death among women who were treated only with HT after undergoing BCS compared with women who were treated with the combination therapy (Table 3).

**Table 3:** All‐cause mortality and radiotherapy plus hormonal therapy use compared to hormonal therapy only

Model	Hazard ratio	95% CI	*P*‐value
Crude	0.217	0.172‐0.273	0.000
Adjusted^a^	0.683	0.508‐0.918	0.012
Propensity score‐matched cohort	0.621	0.458‐0.844	0.002

a Adjusted for age, race, gender, marital status, comorbidities, tumor grade and size, number of node‐positive, year of diagnosis, socioeconomic status, area of residence, and types of hospital (referral vs nonreferral hospital).

Cox‐Snell residual analysis revealed that the hazard function superimposed relatively well with the 45‐degree line except at very large time values (Figure S2), which is common for censored data. As a result, the Cox model was a good fit to the data.

## 4 DISCUSSION

This study aimed to compare real‐world survival rates among patients treated with radiotherapy plus HT vs patients treated with HT only in a cohort of elderly early‐stage HR+ BC patients. Our results showed that radiotherapy plus HT significantly improves the OS of elderly women diagnosed with HR+ BC after PS matching. We controlled for individual, area, and facility‐level information. These findings could inform BC treatment decisions made by the elderly.

The findings of RCTs^1^, ^2^, ^3^, ^4^ have demonstrated the absence of a survival benefit from the addition of radiotherapy after BCS for elderly patients diagnosed with less‐aggressive, early‐stage BC. As a result, the NCCN guidelines support the possible omission of radiotherapy after BCS. In contrast to the findings of RCTs,^2^, ^3^ our study shows that the addition of radiotherapy was associated with improved OS of elderly patients diagnosed with early‐stage HR+ BC. These results were similar to those of other observational studies.^10^, ^11^

A number of factors were negatively associated with OS. Patients aged 80+ at the time of diagnosis had a higher hazard of dying immediately after diagnosis compared with patients diagnosed between the ages of 65 and 69. Older patients were more likely to have more comorbidities compared with relatively younger patients. This is most likely the primary reason why older women exhibited a higher mortality rate than younger women. Our results suggest that patients who have multiple comorbidities have a higher risk of death compared with patients who did not have comorbidities. Like previous studies, we found that the presence of several comorbidities negatively influenced the selection of treatment and OS estimates.^21^, ^22^

The primary advantage of this study relates to the strength of its design. Selection bias was minimized by creating comparable treatment and control groups using PS matching. Additionally, we controlled for individual, facility, and area‐level factors, which contributed toward reducing biases due to unmeasured confounders. Doing so improved the internal validity of this study while maintaining its generalizability.

This study has several limitations. First, we could only adjust for observed covariates.^23^ More sophisticated approaches such as an instrumental variable approach could have been used to control for both observed and unobserved confounders. That being said, we were unable to identify a robust instrumental variable from our dataset. Additionally, physician preferences play an important role in treatment decision and outcomes. However, because our dataset did not include physician information, we could not control for physician preferences. We did not assess BC‐specific survival in this analysis despite it potentially having clinical implications. To our defense, the 5‐year survival rate for women diagnosed with early‐stage BC is approximately 100%, and our data follow the same trend (only 47 out of 5588 elderly women died from BC before PS matching). Patients diagnosed with HR+ and HER2+ patients have a high risk of death compared with patients diagnosed with HR+ with HER2‐ BC. Because SEER registers started collecting HER2 status in 2010, the majority of our data were characterized by an unknown HER2 status. Therefore, we did not assess the effect of HER2 status on the OS of elderly women; we also did not account for HER2 status in the PS matching.

Overall, radiotherapy plus HT was observed to significantly improve the OS of elderly women diagnosed with HR+ BC after PS matching. The results of this study may help inform clinical decision‐making and may serve as a stepping stone for the enactment of cost‐effectiveness analyses to inform resource allocation decision‐making.

## CONFLICTS OF INTEREST

The authors declare no potential conflicts of interest.
