# Supplementary material for: Comparative effectiveness of radiotherapy for early‐stage hormone receptor‐positive breast cancer in elderly women using real‐world data
Source: Cancer Med. 2018 Dec 12;8(1):117–27. doi: 10.1002/cam4.1904 (PMC6346228; doi:10.1002/cam4.1904)
Supplement: Supplementary file 1 [file CAM4-8-117-s001.pdf]

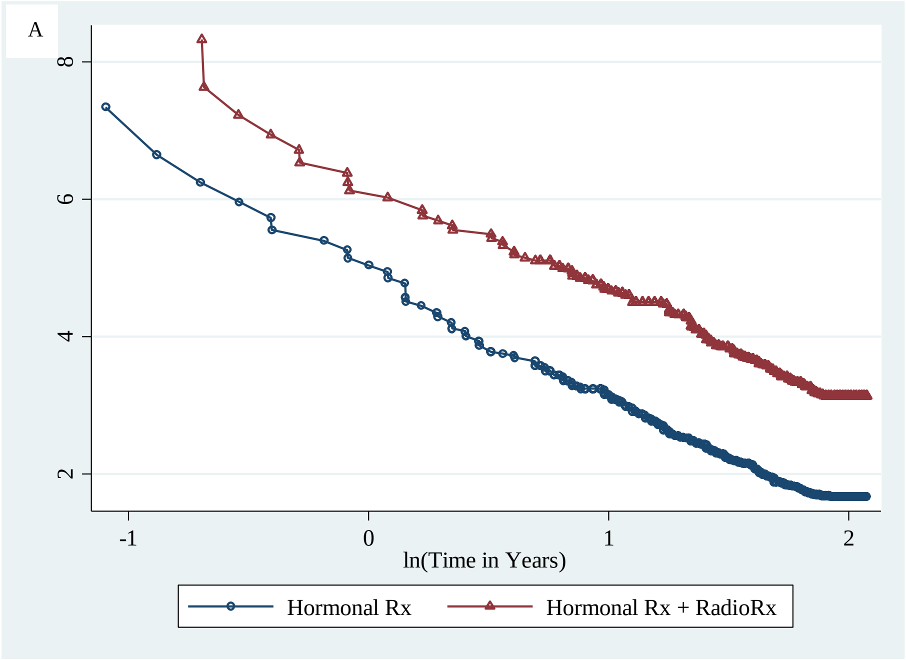

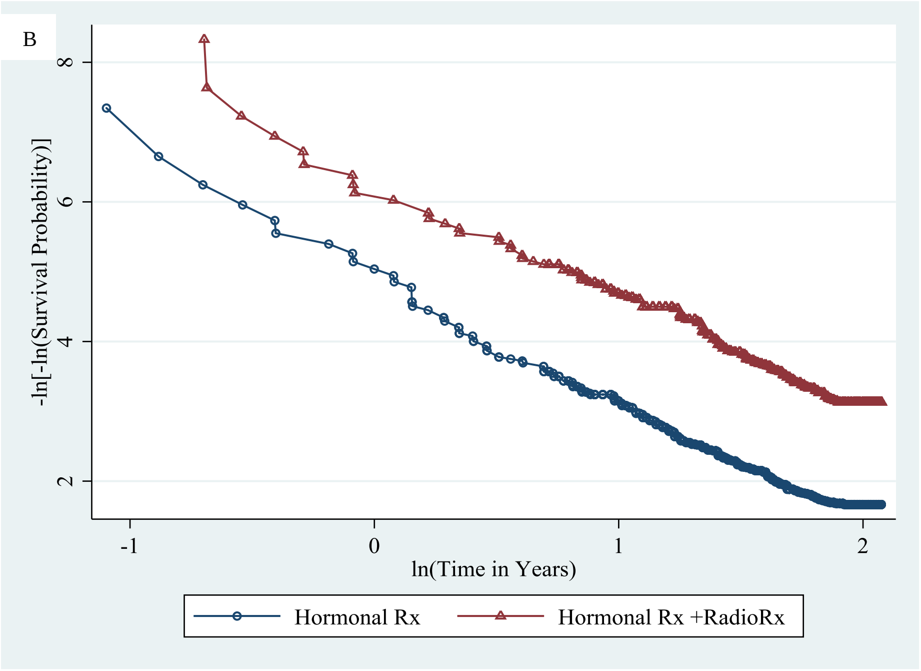

Figure S1 Scaled Schoenfeld residual test by treatment prior to PS matching. A) Scaled Schoenfeld residual test by treatment before PS matching. B) Scaled Schoenfeld residual test by treatment after PS matching. Hormonal Rx + Radio Rx = hormonal therapy plus radiotherapy (red triangles); Hormonal Rx = hormonal therapy alone (blue circles). The *y* axis is log-log of the survival probability, and the *x* axis is log of the survival time in years.
