# Supplementary material for: Comparative effectiveness of radiotherapy for early‐stage hormone receptor‐positive breast cancer in elderly women using real‐world data
Source: Cancer Med. 2018 Dec 12;8(1):117–27. doi: 10.1002/cam4.1904 (PMC6346228; doi:10.1002/cam4.1904)
Supplement: Supplementary file 2 [file CAM4-8-117-s002.pdf]

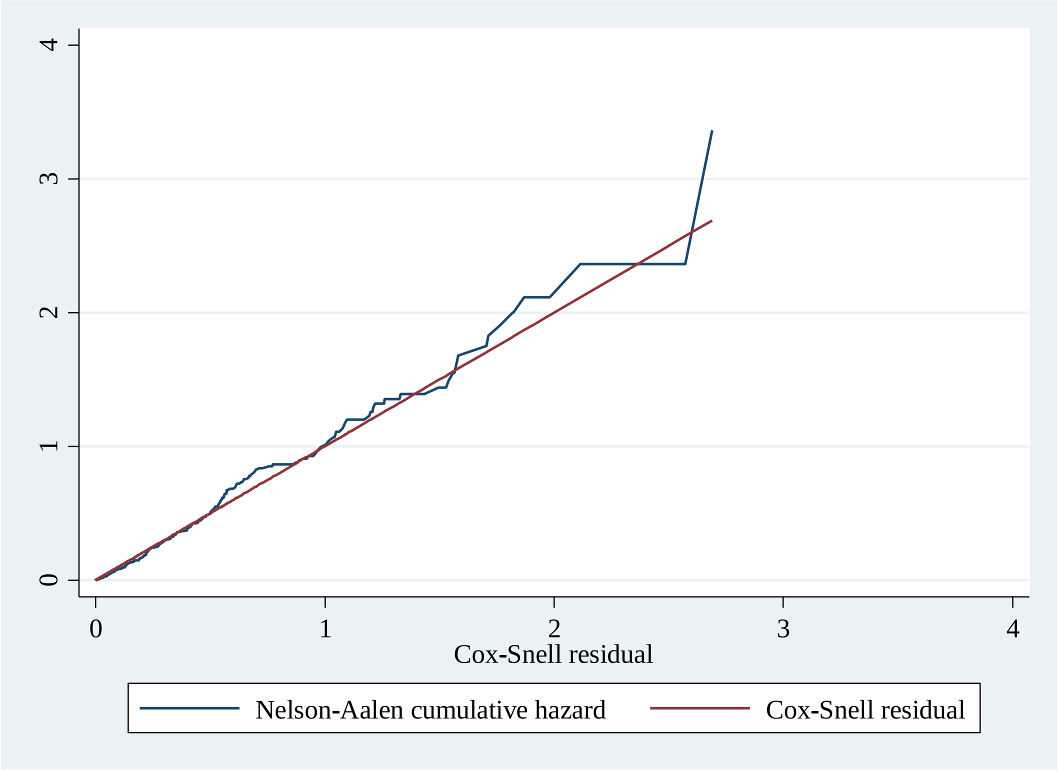

Figure S2 Cox-Snell residuals. The blue line shows the Nelson-Aalen cumulative hazard, and red line shows the Cox-Snell residual.
