# Supplementary material for: Comparative effectiveness of radiotherapy for early‐stage hormone receptor‐positive breast cancer in elderly women using real‐world data
Source: Cancer Med. 2018 Dec 12;8(1):117–27. doi: 10.1002/cam4.1904 (PMC6346228; doi:10.1002/cam4.1904)
Supplement: Supplementary file 3 [file CAM4-8-117-s003.docx]

**Appendix: Online Supplementary**

## Table S1 Treatment identification codes

| Treatments | Healthcare Common Procedure Coding System/ | ICD-9 codes | Revenue code |
| --- | --- | --- | --- |
| BCS | 19110,19120,19125, 19126,19160,19162 | BCS= 8520,8521,8522 8523, 8525 |  |
| Radiation therapy | 76370, 77014, 77261,77262, 7763, 77295, 77299,77300, 77301, 77305, 77310, 77315, 77321, 77331, 77331, 77332, 77333, 77334, 77336, 77338, 77370, 77371, 77372, 77373, 77399, 77470, 0197T, G0338, 77280, 77285, 77290, 61793, 61796, 61797, 61798, 61799, 61800, 63620, 63621, 77401, 77402, 77403, 77404, 77405, 77406, 77407, 77408, 77409, 77410, 77411, 77412, 77413, 77414, 77415, 77416, 77417, 77418, 77419, 77420, 77421, 77422, 77423, 77425--77519, 77520,77521 77522, 77523, 77524, 77525, 77761--77799, 0082T, 0083T, G0173, G0174, G0178, G0242, G0243, G0251, G0339, G0340, 77427,  77431, 7743, 77499 | 92.2, 92.20–92.27, 92.29, 92.3, 92.30–92.39, 92.4, 92.41  v58.0', 'v66.1, v671 | 0330 , 0333 |
| Hormonal therapy (Tamoxifen or Aromatase Inhibitor) | 4179F, 337xF, 3315F |  |  |
| CT scan | 70450, 70460, 70470', 70480-70482, 70486-70488,  70490-70492, 71250, 71260, 71270, 72125-72133,  72192-72194, 73200-73202, 73700–73702, 74150, 74160, 74170, 74176-74178, 76497 | 8703, 8741, 8771, 8801, 8838 |  |
| PET-CT | 78811-78816, 78890, 78891, 78999, G0235, G0253, G0254 | 9211, 9212, 9218, 9219 |  |
| Bone scan | 78300, 78305, 78306, 78315, 78399 | 9214 |  |
| Brain MRI | 70551-70553 | 8891 |  |
| Physician visit | 99201, 99202, 99203,99204, 99205, 99211, 99212, 99213, 99214, 99215 |  |  |

ICD: International Classification of Disease; BCS: breast conserving surgery; ICD-9: International Classification of Diseases, Ninth Revision; CT: Computerized Tomography; PET-CT: positron emission tomography- Computerized Tomography; MRI: Magnetic Resonance Imaging

**Table S2** Baseline characteristics before and after propensity score matching

| Characteristics | Unmatched breast cancer cohort by treatment type | | | Propensity score matched breast cancer cohort by treatment type | | |
| --- | --- | --- | --- | --- | --- | --- |
|  | **No radiation**  **(N = 1,549)** | **Radiation**  **(N = 4,139)** | **p-value** | **No radiation**  (N = 936) | **Radiation**  (N = 936) | **p-value** |
| Age in years, N (%) |  |  | <0.0001 |  |  | 0.8692 |
| Mean age at diagnosis, mean (SD) | 74.81(6.78) | 72.05(5.18) |  | 74.63(6.66) | 74.07(5.77) |  |
| Between 65 and 69 | 416(26.86) | 1600(38.66) |  | 249(26.60) | 250(26.71) |  |
| Between 70 and 74 | 401(25.89) | 1249(30.18) |  | 255(27.24) | 258(27.56) |  |
| Between 75 and 79 | 341(22.01) | 902(21.79) |  | 218(23.29) | 228(24.36) |  |
| 80 and above | 391(25.24) | 388(9.37) |  | 214(22.86) | 200(21.37) |  |
| Race, N (%) |  |  | 0.0024 |  |  | 0.8338 |
| White | 1304(84.29) | 3642(88.08) |  | 790(84.40) | 788(84.19) |  |
| Black | 117 (7.56) | 236(5.71) |  | 67(7.16) | 73(7.80) |  |
| Others | 126(8.14) | 257(6.22) |  | 79(8.44) | 75(8.01) |  |
| Marital status, N (%) |  |  | <0.0001 |  |  | 0.7808 |
| Married | 661(42.67) | 2231(53.90) |  | 503(53.74) | 509(54.38) |  |
| Unmarried | 806(52.03) | 1726(41.70) |  | 433(46.26) | 427(45.62) |  |
| Charlson comorbidity |  |  | <0.0001 |  |  | 0.9182 |
| 0 | 835(53.91) | 2711(65.5) |  | 492(52.56) | 498(53.21) |  |
| 1–3 | 658(42.48) | 1349(32.59) |  | 406(43.38) | 403(43.06) |  |
| ≥ 4 | 56 (3.62) | 79 (1.91) |  | 38(4.06) | 35(3.74) |  |
| Grade, N (%) |  |  | 0.1494 |  |  | 0.8923 |
| Well differentiated | 483(31.18) | 1289(31.14) |  | 287(30.66) | 284(30.34) |  |
| Moderately differentiated | 727(46.93) | 2044(49.38) |  | 481(51.39) | 476(50.85) |  |
| Poorly differentiated and undifferentiated | 255(16.46) | 624(15.08) |  | 168(17.95) | 176(18.80) |  |
| Tumor size, N (%) |  |  | <0.0001 |  |  | 0.9502 |
| 0–2cm | 1151(74.31) | 3358(81.31) |  | 700(74.79) | 694(74.15) |  |
| 2–5cm | 351(22.66) | 669(16.16) |  | 206(22.01) | 211(22.54) |  |
| ≥5cm | 47(3.03) | 112 (2.71) |  | 30(3.21) | 31(3.31) |  |
| Number of positive lymph nodes, N (%) |  |  | <0.0001 |  |  | 0.6984 |
| 0 | 1087(70.22) | 3270(79.06) |  | 670(71.58) | 677(72.33) |  |
| 1–3 | 260(16.80) | 615(14.87) |  | 165(17.63) | 169(18.06) |  |
| ≥4 | 201(12.98) | 251(6.07) |  | 101(10.79) | 90(9.62) |  |
| Year of diagnosis, N(%) |  |  | 0.0241 |  |  | 0.5569 |
| 2006 | 397(25.63) | 1250(30.20) |  | 268(28.63) | 247(26.39) |  |
| 2007 | 243(15.69) | 572(13.82) |  | 155(16.56) | 152(16.24) |  |
| 2008 | 237(15.30) | 602(14.54) |  | 145(15.49) | 140(14.96) |  |
| 2009 | 217(14.01) | 551(13.31) |  | 133(14.21) | 159(16.99) |  |
| 2010 | 237(15.30) | 582(14.06) |  | 135(14.32) | 127(13.57) |  |
| 2011 | 218(14.07) | 582(14.06) |  | 101(10.79) | 111(11.86) |  |
| Community characteristics |  |  |  |  |  |  |
| *Metropolitan area, N(%)* |  |  | *<0.0001* |  |  | *0.8438* |
| Yes | 1272(82.12) | 3758(90.97) |  | 802(85.68) | 799(85.36) |  |
| No | 277(17.88) | 381(9.21) |  | 134(14.32) | 137(14.64) |  |
| Geographic region, N(%) |  |  | <0.0001 |  |  | 0.9279 |
| North East | 293(18.92) | 1055(25.49) |  | 178(19.02) | 167(17.84) |  |
| Midwest | 192(12.40) | 560(13.53) |  | 135(14.42) | 138(14.74) |  |
| South | 496(32.02) | 826(19.96) |  | 256(27.35) | 262(27.99) |  |
| West | 568(36.67) | 1698(41.02) |  | 367(39.21) | 369(39.42) |  |
| Poverty level |  |  | <0.0001 |  |  | 0.8100 |
| 0–5% | 342(22.12) | 1361(32.93) |  | 127(23.18) | 124(22.86) |  |
| 5–10% | 389(25.16) | 1179(28.53) |  | 243(25.96) | 235(25.11) |  |
| 10–20% | 473(30.60) | 1038(24.12) |  | 291(31.09) | 285(30.45) |  |
| 20–100% | 372(22.12) | 555(13.43) |  | 185(19.76) | 202(21.58) |  |
| Referral hospital, N(%) |  |  | <0.0001 |  |  | 0.4556 |
| Yes | 98(6.33) | 156 (3.77) |  | 852(91.03) | 861(91.99) |  |
| No | 1009(65.14) | 2111(51.00) |  | 84(8.97) | 75(8.01) |  |

**Table S3** Absolute standardized differences before and after propensity score matching comparing covariates values for patients receiving radiotherapy plus hormonal therapy and hormonal therapy alone

|  | ASD before matching | ASD after matching |
| --- | --- | --- |
| Age at diagnosis |  |  |
| 65–69 | 0.2534 | 0.0095 |
| 70–74 | 0.0956 | 0.0164 |
| 75–79 | 0.0054 | 0.0295 |
| 80+ | 0.4290 | 0.0566 |
| Race |  |  |
| White | 0.1099 | 0.0143 |
| Black | 0.0746 | 0.0279 |
| Others | 0.0748 | 0.0075 |
| Marital status |  |  |
| Married | 0.2280 | 0.0063 |
| Charlson comorbidity |  |  |
| 0 | 0.2380 | 0.0125 |
| 1–3 | 0.2053 | 0.0042 |
| ≥4 | 0.1043 | 0.0216 |
| Tumor grade |  |  |
| Well differentiated | 0.0084 | 0.0135 |
| Moderately differentiated | 0.0406 | 0.0187 |
| Poorly differentiated and undifferentiated | 0.0440 | 0.0081 |
| Tumor size |  |  |
| <2 cm | 0.1646 | 0.0119 |
| 2–4cm | 0.1648 | 0.0074 |
| >=5 cm | 0.0197 | 0.0118 |
| Number of positive lymph nodes | |  |
| 0 | 0.2043 | 0.0369 |
| 1–3 | 0.0528 | 0.0109 |
| ≥4 | 0.2372 | 0.0407 |
| Year of diagnosis |  |  |
| 2006 | 0.1020 | 0.0538 |
| 2007 | 0.0527 | 0.0141 |
| 2008 | 0.0212 | 0.0058 |
| 2009 | 0.0203 | 0.0774 |
| 2010 | 0.0350 | 0.0209 |
| 2011 | 0.0004 | 0.0326 |
| Geographic regions |  |  |
| North East | 0.1587 | 0.0269 |
| Midwest | 0.0338 | 0.0206 |
| South | 0.2777 | 0.0139 |
| West | 0.0895 | 0.0192 |
| Poverty level |  |  |
| 0–5% | 0.2438 | 0.0124 |
| 5–10% | 0.0381 | 0.0336 |
| 10–20% | 0.1225 | 0.0293 |
| 20–100% | 0.2289 | 0.0357 |
| Referral hospital |  |  |
| Yes | 0.0733 | 0.0406 |
| Metropolitan area |  |  |
| Yes | 0.2556 | 0.0203 |

ASD: absolute standardized difference
